# Relationship between total and differential quarter somatic cell counts at dry-off and early lactation

**DOI:** 10.1371/journal.pone.0275755

**Published:** 2022-10-17

**Authors:** Aldo Dal Prà, Filippo Biscarini, Gian Luca Cavani, Saverio Bacchelli, Alcide Iotti, Sara Borghi, Marco Nocetti, Paolo Moroni

**Affiliations:** 1 Centro Ricerche Produzioni Animali (C.R.P.A.) S.p.A., Reggio Emilia, Italy; 2 Institute of Bioeconomy (IBE), National Research Council (CNR), Florence, Italy; 3 Institute of Agricultural Biology and Biotechnology (IBBA), National Research Council (CNR), Milan, Italy; 4 Albalat, Società Agricola Cooperativa, Modena, Italy; 5 Bonlatte, Società Agricola Cooperativa, Castelfranco Emilia, Modena, Italy; 6 Progeo, Società Cooperativa Agricola, Reggio Emilia, Italy; 7 Università degli Studi di Milano, Dipartimento di Medicina Veterinaria e Scienze Animali, Lodi, Italy; 8 Consortium of Parmigiano Reggiano Cheese, Reggio Emilia, Italy; 9 Quality Milk Production Services, Animal Health Diagnostic Center, Cornell University, Ithaca, NY, United States of America; University of Illinois, UNITED STATES

## Abstract

Mastitis is a most common disease of dairy cows and causes tremendous economic loss to the dairy industry worldwide. Somatic cell counts (SCC) reflect the inflammatory response to infections and is a metric used as key indicator in mastitis screening programs, typically within the framework of national milk recording schemes. Besides the determination of total SCC, the differentiation of cell types has been described to be beneficial for a more definite description of the actual udder health status of dairy cows. Differential somatic cell count (DSCC) represents the combined proportion of polymorphonuclear leukocytes (PMN) and lymphocytes expressed as a percentage of the total. The aim of this study was to investigate the relationship between SCC and differential somatic cell count (DSCC) in individual quarter milk samples collected at different time points: at dry-off, after calving and at the lactation peak. We used individual quarter data from farms representing the specialized production system of Parmigiano Reggiano cheese in Northern Italy. Average DSCC values ranged between 44.9% and 56.3%, with higher values (60.4%-72.1%) in milk samples with ≥ 1 million SCC/ml (where the proportion of samples with DSCC > 70% can be as high as 0.73). Moderate overall correlations between DSCC and log(SCC) were estimated (Pearson = 0.42, Spearman = 0.38), with a clear increasing trend with parity and around the lactation peak (e.g. Pearson = 0.59 at 60 DIM in parity 4). Taking SCC values as indicators of subclinical mastitis, DSCC would diagnose mastitis with 0.75 accuracy. Data editing criteria do have an impact on results, with stricter filtering leading to lower correlations between log(SCC) and DSCC. In conclusion DSCC and SCC provide different descriptions of the udder health status of dairy cows which, at least to some extent, are independent. DSCC alone doesn’t provide more accurate information than SCC at quarter level but, used in combination with SCC, can be of potential interest within the framework of milk recording programs, especially in the context of selective dry-cow therapy (SDCT). However, this needs further investigation and updated threshold values need to be selected and validated.

## Introduction

Mastitis is one of the most prevalent and costly diseases in the dairy cattle industry worldwide and adversely affects the welfare of dairy cows [1]. Intramammary infections (IMI) lead to a reduction of milk quality and quantity and an increased use of antibiotics. The negative effects of clinical and subclinical mastitis are also related to the persistence and recurrence of infection. The possibility of using bacterial culture results to control and reduce IMI is a viable option in some circumstances. However, laboratory accessibility and the costs for standard or on-farm culture represent a serious obstacle for some farms [2, 3].

Somatic cell counts (SCC) provide an indication on the inflammatory response in the mammary gland and, in association with bacterial culture, are a proxy for identifying and monitoring IMI and milk quality at quarter, cow, herd, and population levels [4]. Currently, the most practical and sustainable way to manage the udder health in dairy herds is by monitoring individual SCC. This method does not have the same accuracy as microbiological analysis because SCC can only suggest the presence or absence of inflammation.

The usefulness of SCC in monitoring the udder health has been studied intensively and thresholds to distinguish between uninfected and infected quarters have been suggested, including recommendations for the use of repeated SCC measurements (e.g. [4–6]). There is a consensus on the association between an increase in SCC in milk with a change in the proportion of inflammatory cells in the milk cell population, given that the increase in cell numbers is mainly due to the migration of PMN (polymorphonuclear leukocytes) from blood circulation to milk [7, 8].

The recent possibility of using a milk analyser (Fossomatic™7DC, Foss A/S, Hilleød, Denmark), able to perform a DSCC (differential SCC), fully integrated with DHIA (Dairy Herd Improvement Association) instruments currently used to analyse individual milk samples, may open new opportunities to apply DSCC as a tool to identify mastitis in combination with SCC. Differential SCC has recently been described as a new tool for monitoring udder health ([9–12]), and the methodology for simultaneous determination of SCC and DSCC has been published [13]. Alongside the determination of SCC, DSCC has been described to be beneficial for a more definite description of the actual udder health status of dairy cows. A recent study [14], evaluated DSCC as an additional indicator of SCC for the identification of IMI in dairy cows at the end of the lactation period. The DSCC targets the three main types of immune cells in milk: polymorphonuclear neutrophils (PMN), lymphocytes, and macrophages, and represents the combined proportion of PMN and lymphocytes expressed in percentage. The proportion of macrophages can be calculated by subtracting DSCC from 100%.

The main objective of this study was to further characterize the relationship between SCC and DSCC in milk, specifically in the context of a specialized dairy production system, that of Parmigiano Reggiano PDO cheese in Northern Italy, from dry-off to the lactation peak. Secondarily, we wanted to understand whether, under field conditions, DSCC can potentially add value to per-quarter SCC measurements for the identification of mastitis in dairy cows without the support of bacteriology, especially in the context of selective dry-cow therapy (SDCT). As a matter of fact, in the farm practice the use of milk cultures presents limitations, i.e. the cost-benefits ratio, the timeliness of results and the labour-intensity. To these aims, individual quarter milk samples were collected at dry-off (T1), after calving (2–9 DIM –days in milk– T2) and at 60–77 DIM (T3). Model-based phenotypic correlations between SCC and DSCC were estimated at each time point on the whole dataset and stratified by cow parity and SCC class (from 50,000 scc/mL to more than 1,000,000 scc/mL), to fully characterize the relationship between these two metrics of inflammatory response in the mammary gland.

## Materials and methods

### Ethical statement

The research protocol was part of an agreement by the Italian Ministry of Health (authorization n. 628/2016-PR) with the University of Milan, and methods were carried out in accordance with the approved guidelines. Milk samples used for the analyses were collected by farm technicians for standard Parmigiano Reggiano routine recording and in accordance with specific hygiene requirements. Cows were not subjected to any invasive procedures.

### Herd selection

All dairy cows involved in this study were reared on 3 farms in Northern Italy (region Emilia Romagna) within an established collaboration with the “Dipartimento di Medicina Veterinaria” of the “Università degli Studi”, Milan, Italy. One of the selection criteria was the use of SCC and DSCC testing under DHIA monthly control. Farms A and C are in Castelfranco Emilia (N 44°61′87.2″ E 11°11′85.7″ and N44°59′33.9″ E11°12′74.0″), and Farm B in Modena (N44°68′78.9″ E10°97′90.3″). From December 2018 to June 2020, for a period of at least one lactation (including dry-off), 17512 quarter milk samples were collected from 1830 different cows. In farms A (760 lactating cows), B (935) and C (640) cows belonged to the following breeds: 100% Italian Holstein-Friesian (Farm A) and crossbreed PROcross™(cross breeding system) cows (88% B and 90% C). Cows were located in pens with separate solid slurry and milked twice per day in conventional milking parlours. A TMR (total mixed ration) mainly consisting of Italian ryegrass, alfalfa hay, cereals and concentrates was fed to all cows according to the Protected Designation of Origin (PDO) cheese Parmigiano Reggiano production regulations (Table 1). The average herd milk yield ranged between 8631 and 9025 kg/yr with 3.39–3.79% fat and 3.49–3.60% protein. The average number of parity and DIM varied from 2.4 to 2.6 and from 132 to 159, respectively. Bulk tank-milk average SCC was 159,000 scc/mL (farm B), 210,000 scc/mL (farm A) and 290,000 scc/mL (farm C).

**Table 1:** Characteristics of herds considered in the current study.

Herd	A	B	C
Lactating cows	760	935	640
Breed (Lactating)	Italian F Holstein-Friesian	crossbreed^™^PROcross 88% Italian F Holstein-Friesian 12%	crossbreed^™^PROcross 90% Italian F Holstein-Friesian 10%
Milking Parlour	Rotary	Rotary	Herringbone
Feeding (kg of DM)	Alfalfa hay, 5.33	Alfalfa hay, 10	Alfalfa hay, 6.1
	Italian ryegrass hay, 3.7	Italian ryegrass hay, 3	Italian ryegrass hay, 2.9
	Soy meal, 0.9	Corn meal, 4	Soy meal, 0.9
	Wheat meal, 3.5	Sorghum meal, 2	Wheat meal, 3.5
	Sorghum meal, 5.3	Barley meal, 2	Sorghum meal, 5.3
	Barley meal, 1.8	Liquid molasses, 1	Barley meal, 1.8
	Concentrate, 6.2	Concentrate, 3	Concentrate, 6.2
	Vitamin and mineral, 0.1	Vitamin and mineral, 0.1	Vitamin and mineral, 0.1
Milk destination	Parmigiano Reggiano	Parmigiano Reggiano	Parmigiano Reggiano
Average milk production kg/d	30.5	30.5	28.1
Average bulk tank somatic cell count (1000⋅ scc/mL)	210	159	290

DM: dry matter.

### Study design, enrolment and sampling

Cow enrolment began in December 2018 and continued until June 2020. Milk samples were collected at 3 different time points -at dry off (T1: 3–4 days before dry-off, after the previous lactation), within 2 to 9 DIM (T2), and within 60 to 77 DIM (T3)- by on-farm trained personnel within each herd, with training and supervision conducted by the principal investigators. Cows eligible for enrolment had no record of receiving parenteral or intramammary treatment with an antibacterial or anti-inflammatory medication during the 30 days immediately before dry-off and showed no signs of clinical mastitis at dry-off. Cow were checked for the absence of previous treatments. During the dry-off period, all quarters were sealed with internal teat sealants. Quarters with SCC > 200,000 scc = mL and/or DSCC > 70% were additionally treated with antibotics. During the study, milk production, fat, protein, SCC and DSCC were recorded through monthly DHIA testing in all herds. Cow data were collected using a computerized herd record keeping system Afifarm (TDM s.r.l., San Paolo, Brescia, Italy).

### Determination of SCC and DSCC

Quarter milk samples were obtained from farm technicians and each quarter was cleaned with chlorhexidine before sampling. First streams of foremilk were discarded, and then 50 mL of milk were collected aseptically from each quarter into vials. Samples were stored at 4°C until SCC and DSCC tests were performed using the Fossomatic^™^7DC (Foss A/S, Hillerød, Denmark). The analysis of DSCC was based on Foss DSCC Method Cell Staining (international patent PCT/EP2010/065615- Holm, 2012), which allows identification within a milk sample of the macrophages (MAC) and the combination of PMN (polymorphonuclear neutrophils) and LYM (lymphocytes). The DSCC is expressed as the combined proportion (%) of PMN and LYM on the overall count of milk cells.

### Data processing and statistical analysis

We received a dataset on 1830 cows for a total of 17512 quarter milk samples and removed records with missing SCC or DSCC data, records with parity lower than 2 and from cows with fewer than 3 records. This left 15598 records from 1638 cows available for the analysis. For the linear correlation analysis, SCC data were log-transformed (natural logarithm, linear score [15]). The data were collected on a per-quarter basis: the observation units were individual quarter records, which were hierarchically structured within individuals and repeated over time. Observations could not be assumed to be independent from each other but were correlated within individual cows. This was taken into account in the bivariate linear mixed models used to estimate the adjusted correlations between log(SCC) and DSCC, in the whole dataset (overall) and stratified by i) parity, ii) timepoint, iii) parity and timepoint:

$$\begin{array}{c} \begin{array}{c} {\text{y}}_{iktpjmz}=\mu +{\text{herd}}_{k}+{\text{timepoint}}_{t}+{\text{parity}}_{p}+{\text{treatment}}_{j}+{\text{breed}}_{m}\\ +\text{DIM}+\text{MY}+{\text{cow}}_{z}+{\text{e}}_{iktpjmz} \end{array} \end{array}$$ (1)

where y_*iktpjmz*_ is the log(SCC) or DSCC value for record *i* from cow *z* belonging to herd *k*, parity *p*, breed *m* at timepoint *t* with treatment *j* (teat sealant or teat sealant + antibiotic); *μ* is the intercept, DIM are the days in milk and MY is the milk yield (kg); cow_*z*_ is the random effect of the individual cow, and e_*iktpjmz*_ is the residual. The same model was used for both traits, which in matrix notation becomes:

$$\begin{array}{c} y=\mu +\text{Xb}+\text{Zu}+e \end{array}$$ (2)

with **y** = [**y**_**1**_, **y**_**2**_], vector of phenotypic values for the two traits (SCC and DSCC), ***μ*** = [*μ*_1_, *μ*_2_], vector of the intercepts for the two traits; **b** = [**b**_**1**_, **b**_**2**_], vector of systematic effects for trait 1 and trait 2, **u** = [**u**_**1**_, **u**_**2**_] vector of random cow effects for both traits; **e** = [**e**_**1**_, **e**_**2**_] vector of residuals. **X** and **Z** are design matrices that relate records to systematic effects (**X**) and to cow effects (**Z**_(*n*,*z*)_: *n* rows, one per record, and *z* columns, one per cow with 1’s corresponding to records belonging to each cow, 0’s otherwise).

The (co)variance structure included a variance component for the cow effect, plus residual variance and covariance components:

$$\begin{array}{c} Var\left(y\right)=\text{RI}\sigma_{u}^{2}+\text{RI}\sigma_{e}^{2} \end{array}$$ (3)

where **R** is the residual variance and covariance matrix between traits; **I** is the identity matrix; $\sigma_{u}^{2}$ and $\sigma_{e}^{2}$ are the cow (permanent environment) and residual variances.

Data processing and statistical analysis were performed with the R environment for statistical programming [16]. Specifically, the *sommer* [17] and *tidyverse* [18] packages were used.

## Results

### Data description

Overall, as indicated in Table 2, 15598 quarter milk samples were collected from a total of 1638 cows in 3 dairy herds (A = 437, B = 708 and C = 493) with a parity average of 2.3 lactations (A = 2.3, B = 2.3 and C = 2.4). Average dry-off period was 63 days (A = 53, B = 62 and C = 75) and length of lactation was 319 DIM (A = 352 DIM, B = 312 DIM and C = 297 DIM). SCC were high if compared with the farm milk quality standard: average at dry-off (T1) was 576,000 scc = mL (A = 708,000 scc/mL, B = 461,000 scc/mL and C = 621,000 scc/mL); at T2 the average was 259,000 scc/mL (A = 198,000 scc/mL B = 273,000 scc/mL and C = 293,000 scc/mL); at T3 the average was 237,000 scc/mL (A = 273,000 scc/mL, B = 120,000 scc/mL and C = 424,000 scc/mL). As for DSCC, at T1 it was 54% (A = 56% B = 53% and C = 53%), at T2 it was 50% (A = 51% B = 49% and C = 50%) and at T3 it was 47% (A = 50% B = 45% and C = 49%).

**Table 2:** Descriptive statistics of the farms enrolled in the study.

Parameter	Farm A	Farm B	Farm C	total
Number of records	3845	7486	4267	15598
Number of cows	437	708	493	1638
Parity (avg)	2.3	2.3	2.4	2.3
Milk Yield (avg)	8868.8	8813.9	8812.7	8831.8
Dry period length (avg)	53.1	61.9	74.6	63.2
DIM T1 (avg)	352.5	311.7	297.3	320.5
DIM T2 (avg)	11.4	4.3	7.6	7.8
DIM T3 (avg)	64.8	60.4	77.5	67.6
SCC T1 (avg)	707.9	460.9	621.1	596.6
SCC T2 (avg)	197.9	273.5	293.0	254.8
SCC T3 (avg)	273.5	120.0	424.3	272.6
DSCC T1 (avg)	56.3	52.8	53.0	54.0
DSCC T2 (avg)	50.9	49.1	50.2	50.1
DSCC T3 (avg)	50.0	44.9	48.6	47.8

Milk yield: kg/cow/lactation; DSCC: %; SCC: 1000*cells/ml; Dry period length: n. of days.

### Correlations between SCC and DSCC

**Fig 1:**
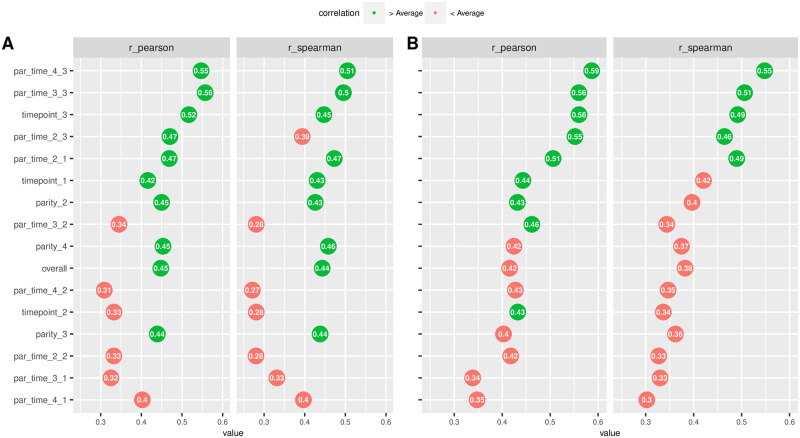
Raw and model-based correlations. A) raw correlations (Pearson, left and Spearman (rank), right) between log (SCC) and DSCC. B) model-based correlations (Pearson, left and Spearman (rank), right) between log (SCC) and DSCC. Overall (whole dataset) and stratified (parity, timepoint, and timepoint-by-parity: e.g., par_time_2_3 indicates parity 2 and timepoint T3) correlations. Green and red colors indicate whether specific correlations are above or below average.

In Fig 1, panel (A), we report the ‘raw’ (not model adjusted) Pearson (linear) and Spearman (rank) correlations between log(SCC) and DSCC. Overall correlations were moderate (0.488 and 0.443). Stratification showed an increase with parity and around the lactation peak, with the highest correlations found in parity 4 and at T3 (Pearson = 0.547, Spearman = 0.505). When adjusting for systematic effects (Fig 1, panel (B)), correlations were generally higher ranging from a minimum for parity 4 at T1 (Pearson = 0.35, Spearman = 0.30) to a maximum for parity 4 at T3 (Pearson = 0.59, Spearman = 0.55). Fig 1 clearly shows an increasing trend around the lactation peak (T3). The trend for parity is less clear, with higher correlations at parity 2 and 4, and relatively lower values at parity 3.

**Fig 2:**
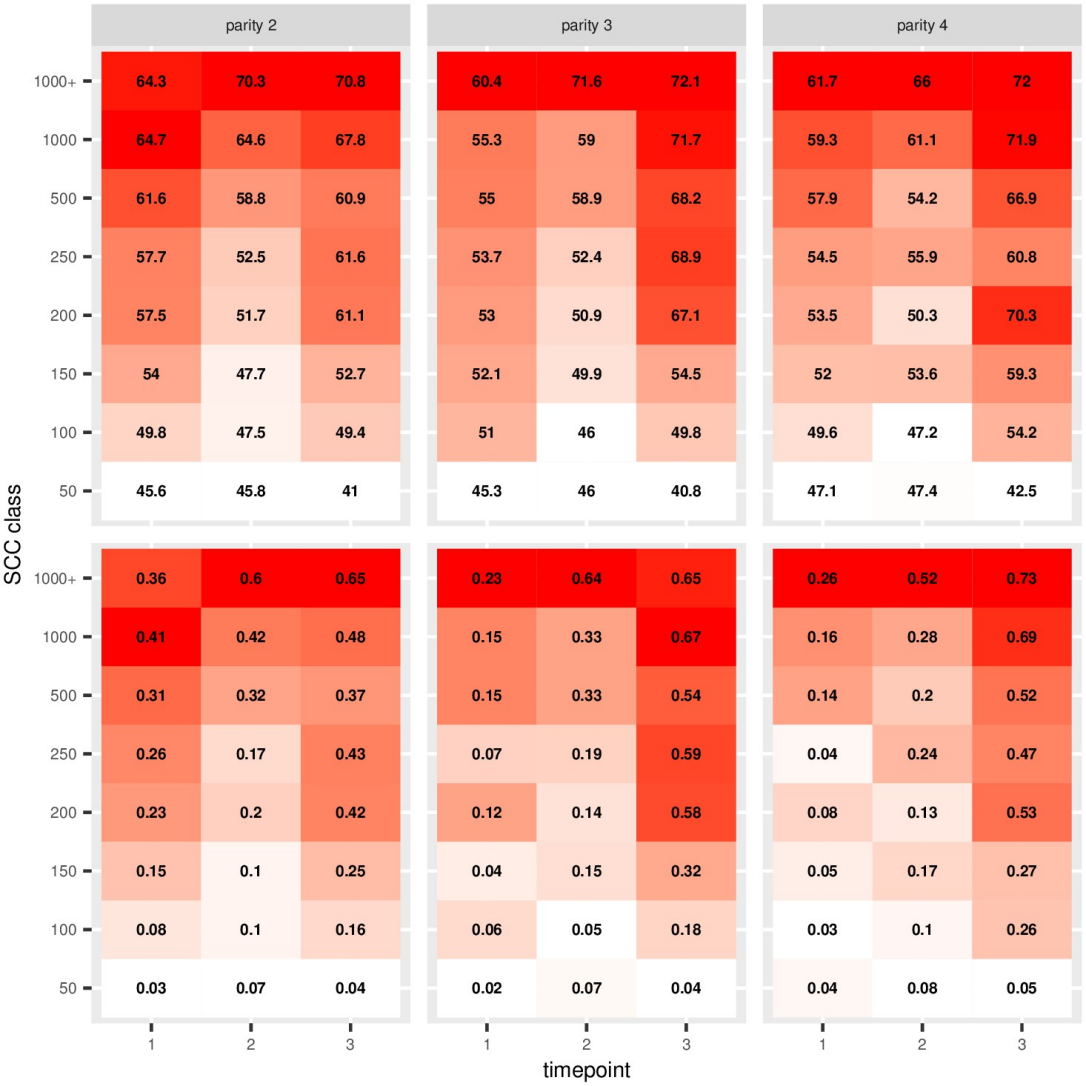
DSCC along SCC classes. Average DSCC per SCC class, parity and timepoint (above), and proportion of records/samples with DSCC > 70% (below). Darker colors correspond to higher values.

The recently introduced DSCC metric is generally seen as complementary to the well-established SCC. In this study we investigated the average DSCC per SCC class overall and stratified by parity and/or timepoint, and also the proportion of records with DSCC >70% (Fig 2, S1 Table). When SCC increases (SCC classes from 50,000 scc/mL to 1000000+ scc/mL), there is a clear trend towards increasing average DSCC (Fig 2, top) and increasing proportions of DSCC values >70% (Fig 2, bottom). These trends are consistent across parities and timepoints, but the increase is slower around calving (T2), when both average DSCC and proportion of DSCC >70% remain stable over higher values of SCC.

## Discussion

### Relationship between SCC and DSCC

Many indicators for mastitis have been developed and investigated over the last half century [19, 20]. SCC, typically determined on highly accurate high-throughput analyzers, has become the key parameter in routine mastitis monitoring programs within the framework of DHI testing since its introduction in the early 1980s [13]. In Italy, among the diseases that affect dairy cows, mastitis is still the most important and significantly affects the profits of dairy farmers [21]. Several studies [8, 22] revealed merit in differentiating milk immune cells in addition to determining the total SCC for a finer description of the udder health status of dairy cows. The new Foss DSCC method can indicate IMI during lactation and at dry off and may react differently between IMI with major and minor pathogens [23, 24]. According to Halasa and Kirkeby [25], it is important that future research focuses on conducting field studies comparing management practices with and without using the DSCC, to validate the usefulness of this parameter under field conditions and allow translating research findings into commercial applications (for details and benchmark methods see [26]). This would aid in the establishment of general guidelines for the use of DSCC to improve udder health in dairy cattle herds. To support this idea, part of the aim of this work was to estimate phenotypic correlations, at quarter level, between DSCC and SCC in quarter milk samples at 3 different time points. Both Pearson (linear) and Spearman (rank) correlations were always positive, indicating that higher SCC values correspond to a prevalence of PMN and LYM (higher DSCC). Adjusting for systematic effects and data structure led to generally higher correlation estimates compared to raw unadjusted correlations, probably because of removing background noise. Within parity, timepoint and SCC levels different correlation trends have been observed, with generally higher correlations at later parities, around lactation peak (T3) and with higher SCC counts. As a consequence of stratification of the analysis, some of the reported correlations are based on a relatively smaller number of records, e.g. at T2 and T3 in parity 4: however, also in these extreme cases, still 922 and 744 records were used.

The average DSCC values observed in this study are generally lower than those reported in literature, although broad variations have been observed (34–79%) for DSCC in milk samples [13, 26, 27]. Previous studies showed that DSCC are influenced by DIM and parity, but in a different way compared to SCC. SCC generally decreases over the lactation, while for DSCC the reported results are inconsistent: some suggested it increases with DIM [14], some suggested it decreases with DIM [23]. A dilution effect has been suggested to explain the decrease in SCC [28, 29]; DSCC seems to be less affected by this dilution effect as it clearly follows the lactation curve.

In S1 Table and in Fig 2 we analyzed the average DSCC per SCC class (from < 50 000 to > 1 00 0000 scc/mL) overall, per parity and timepoint. In quarters that are considered healthy based on SCC (50 000 to 200 000 scc/mL) the DSCC increased from 44% to 55% in the entire dataset, although DSCC as high as 67% or 70% could be observed depending on parity and stage of lactation (parity 3 and 4, lactation peak -T3). It should be noted that in terms of DSCC, results from samples with < 50 000 scc/mL may be less reliable (e.g. poor repeatability) as they are out of the performance range of the described detection method [13]. When we considered subclinically infected quarters (> 200 000 to more than 1 000 000 scc/mL), DSCC increased from 56% to 65% with peak values at 70–72% at T2 (calving) and T3 (peak lactation), but with little differences between parities.

### DSCC as a tool for detecting subclinical mastitis

**Fig 3:**
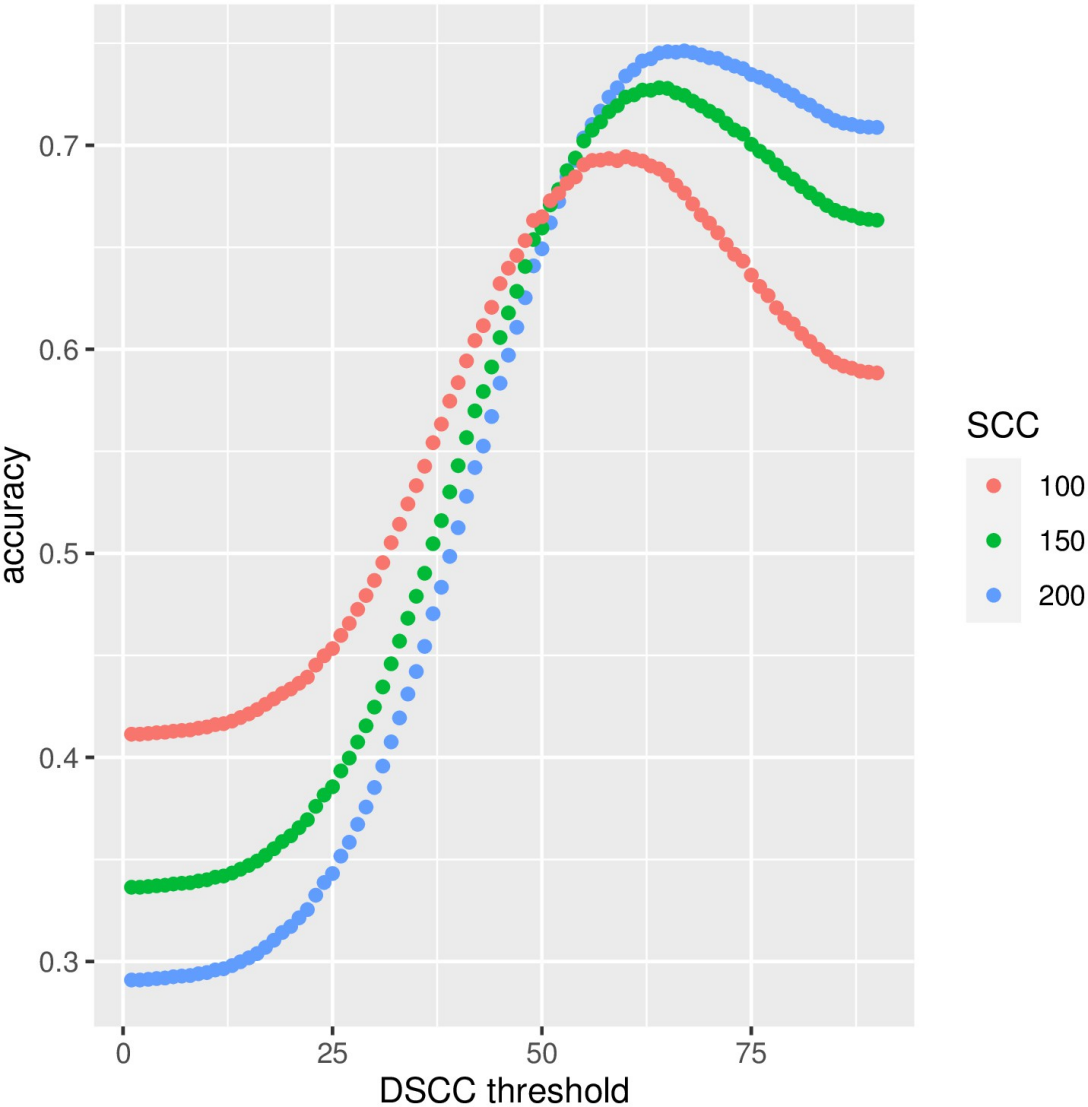
SCC vs DSCC for IMI detection. Total correct predictions (subclinical mastitis: yes or no) based on DSCC values. Subclinical mastitis to be predicted was defined based on three different SCC thresholds: > 200k, > 150k, > 100k.

In dairy farm practice, SCC is usually used as criterion to diagnose subclinical mastitis (IMI) with threshold > 200, 000 scc/mL; however, at different levels below the 200,000 scc/mL threshold there can also be IMI. Proposals to replace or complement SCC-based diagnosis of IMI with DSCC have been put forward, with proposed DSCC thresholds ranging between 65% to 72% [13]. Taking SCC as a reference at thresholds 100, 000 scc/mL (red line), 150,000 scc/mL (green line) and 200,000 scc/mL (blue line), we show in Fig 3 the predictive accuracy of using DSCC alone to diagnose IMI. The highest accuracy would be 0.75 at DSCC = 67%, when the SCC > 200k/ml is used as criterion for the “true” subclinical mastitis. This is obviously not a formal attempt at building a predictive model for IMI, and DSCC is likely to be better used in combination with SCC (and other parameters, e.g. linked to milk production and the lactation cycle) in one of several possible statistical models (see for instance [30]). The results shown in Fig 3, though, provide further insights into the relationship between SCC and DSCC and offer a basis upon which DSCC can be used for its potential contribution to the predictive ability of subclinical mastitis in combination with other parameters. The fact that DSCC does not perfectly reproduce SCC results in terms of the detection of subclinical mastitis, together with the lower than 1 correlation between these two metrics, may actually indicate that there is room to use DSCC together with SCC for improved farm applications for the monitoring of udder health. This may be particularly useful in the case of selective dry-cow therapy (SDCT) to help identify the animals to treat.

On the basis of our data, DSCC alone does not provide sufficient information but must be used together with the total somatic cell value to assess accurately the health status of the cow [4, 26]. With 200,000 scc/mL the cow is considered infected if the DSCC has a percentage > 60% (sensitivity 67–87%) according to Schwarz et al. [14]. Zecconi et al. in 2019 [27], with the same approach, identified three DSCC values based on days in milk (DIM): 66.3% (< 100 DIM), 69.2% (101–200 DIM) and 69.3% (> 200 DIM) (81% accuracy and 67% sensitivity). If only one of the two somatic or differential cell values is greater than the threshold values indicated, the cow can be at risk of mastitis or be in a chronic disease state. In our study we didn’t find a strong correlation between low SCC and low value of DSCC, like Stocco et al. [31] in composite samples, and the analysis of data showed, as in a previous work [27], that DSCC has not consistent performances, confirming the presence of confounding factors such as parity and days in milk.

Despite our results showing how the correlation between SCC and DSCC increases with parity, we need to take in consideration that the average number of lactations in Italy is currently 2.4±0.77 lactations. Less than 10% of the Italian dairy farms have average parity (number of lactations) >3 (Italian Breeders Association, relating to the Italian Holstein-Friesian breed, 2021). This aspect, in relation to the results of our study, could lead to a worsening of predictive ability of subclinical mastitis using DSCC in combination with SCC on the Italian dairy farms (e.g. 2x2 screening tables as proposed by Zecconi et al. [27]: see S1 Fig for an illustration with data from the present study). This however, needs to be demonstrated experimentally with milk culture data. In any case, the DSCC thresholds suggested in literature [13] should probably be adapted to different farming contexts and management (e.g. breed, parity) and technology related to milk production.

### Data editing criteria

**Fig 4:**
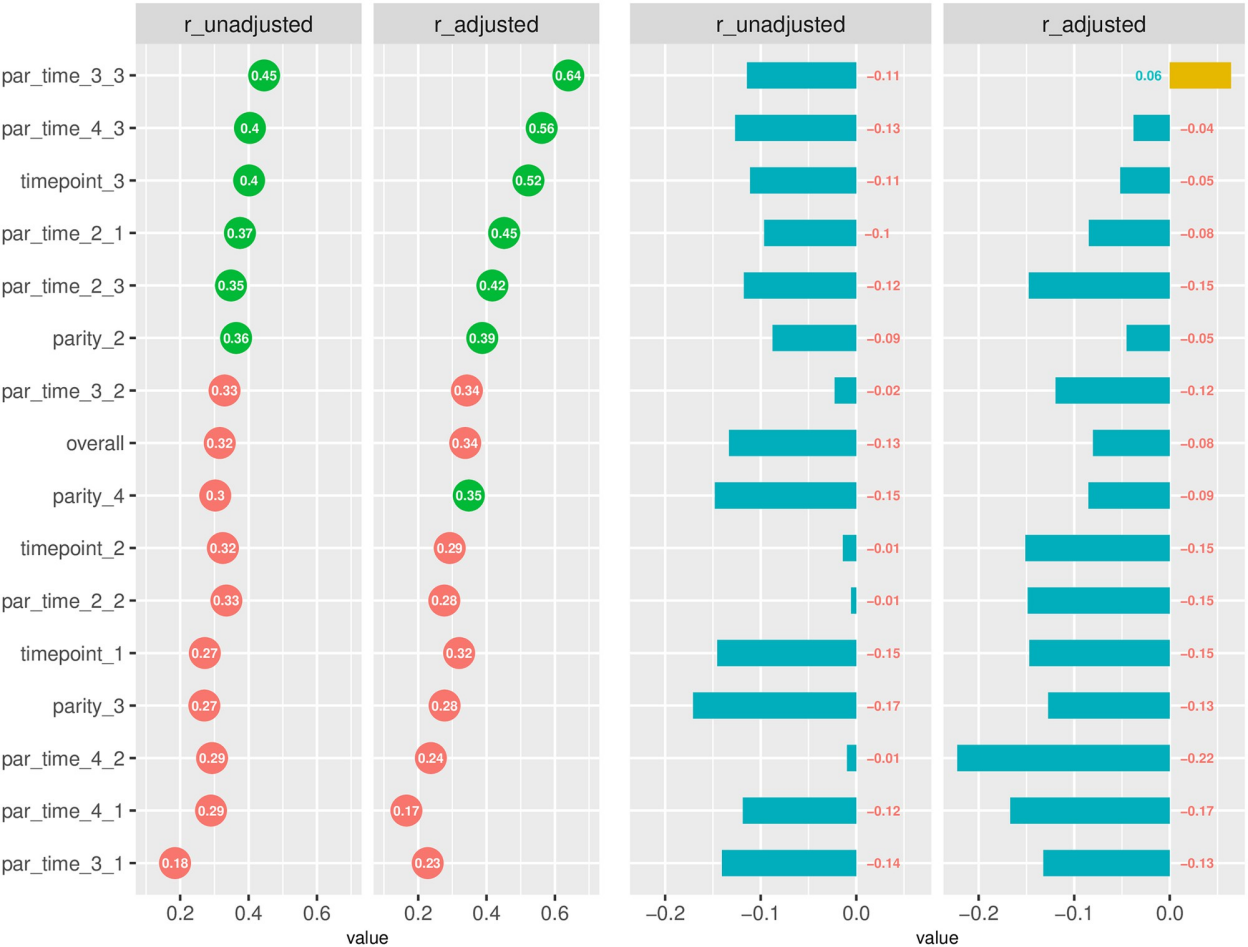
SCC vs DSCC for IMI detection. Pearson correlations between DSCC and log (SCC) when applying the editing criteria for SCC recommended in Damm et al, 2017 (SCC in [50k, 1.5M]. On the left, unadjusted and adjusted (model-based, equations (1,2,3)) correlations: green/red circles represent correlation coefficients above/below the average. On the right, differences with correlations estimated from the editing criteria applied in this study (SCC in [1k,10M]): negative/positive values represent smaller/larger values compared to the results from the present study. The comparison has been normalised by size (same data size in both sets of calculations).

Damm et al. [13] in their founding work on DSCC proposed to use only SCC records between 50000 and 1.5 million scc/mL, claiming these would translate into reliable and robust DSCC estimates. We used SCC records with values lower than 10 million scc/mL, following the standard cut-off values applied in the national dairy recording scheme. However, we asked ourselves what our results would look like if we followed the same data editing criterion as in Damm et al. [13]. Would the relationship between SCC and DSCC be different? After removing records with SCC < 50, 000 scc/mL or SCC > 1,500,000 scc/mL, 7688 records from 1584 cows were left. Fig 4 shows results from the comparison, in terms of DSCC-log(SCC) Pearson correlations (unadjusted and model-adjusted), between the editing for SCC used in this study (SCC in [1 000–10 000 000 scc/mL]) and that used in Damm et al. 2017 (SCC in [50,000 scc/ml and 1,500,000 scc/ml]). The comparison was made after normalizing data by size: since 7688 records were left when editing out SCC values lower than 50,000 scc/ml and higher than 1.500,000 scc/ml, the same number of records was subsampled from the 15598 records with SCC < 10,000,000 scc/ml. Subsampling was performed randomly and unstratified, without replacement. To avoid potential biases linked to specific subsamples of the original data, this subsampling was repeated 10 times, and averages have been presented as results. We see that correlations between log(SCC) and DSCC, both unadjusted and model-adjusted, are consistently lower when tightening the filtering criteria and allowing a narrower range of data in the calculations. This may be partly linked to the removal of outliers (milk samples with exceptionally low or exceptionally high SCC values) which, when included, would pose a challenge to the estimation of linear correlations, largely based on the assumption of normal distribution of the data. A lower correlation between SCC and DSCC when SCC is low has been reported previously [14]. The fact that the estimation of phenotypic correlations appears to be sensitive to the range of SCC data used will likely have consequences on the predictive ability of DSCC (alone or in combination with SCC and/or other milk parameters) and on its applications for routine management of dairy herds. The practical application of DSCC in the frame of DHI testing programs needs therefore further investigation.

## Conclusion

DSCC has recently emerged as an additional metric that can be used to monitor udder health in dairy cows. In this work, we analysed the relationship between DSCC and SCC, the potential use of DSCC for the management of subclinical mastitis, and the impact of data editing criteria on the results.

The availability of this new tool allows to improve the management of subclinical mastitis without resorting to bacterial culture, and could be useful in the context of SDCT to identify the animals to treat. Our results show that DSCC and SCC are moderately correlated, and that their correlation changes with parity and the lactation cycle, being higher in older cows and around the lactation peak. By providing a partially independent source of information, DSCC is potentially a suitable candidate to be used together with SCC to increase the accuracy of the diagnosis of subclinical mastitis in dairy cows at different lactation stages (specific DSCC thresholds need to be identified, though). Evaluating leukocytes in proportion and absolute values can provide a more complete overview of the immune system reaction to IMI, possibly in relation to specific causative pathogens, and detect stronger relationships with SCC.

This study based on data derived from a large dataset across three dairy farms is an important contribution towards the development of predictive models for subclinical udder infections, which could be used for the management of dairy cows. The practical application of DSCC at dry-off and early lactation could be interesting within the framework of DHI testing programs but needs further investigation and more precise alert thresholds need to be selected and validated also in relation to the specific management of dairy farms.

## Supporting information

S1 Table DSCC along SCC classes. Average DSCC per SCC class (horizontal axis: from 50k to > 1000k) overall and per parity or timepoint, in the top section of the table; proportion of records with DSCC >70% in the bottom section of the table.
(PDF)

S2 Table Estimated fixed and random effects from the DSCC-SCC bivariate model. (PDF)

S1 Fig 2x2 tables with the distribution of samples along combinations of SCC and DSCC above/below critical thresholds. (PDF)
