# Supplementary material for: Relationship between total and differential quarter somatic cell counts at dry-off and early lactation
Source: PLoS One. 2022 Oct 17;17(10):e0275755. doi: 10.1371/journal.pone.0275755 (PMC9576081; doi:10.1371/journal.pone.0275755)
Supplement: S1 Table — Average DSCC per SCC class (horizontal axis: from 50k to > 1000k) overall and per parity or timepoint, in the top section of the table; proportion of records with DSCC >70% in the bottom section of the table. (PDF) [file pone.0275755.s001.pdf]

Relationship between total and differential quarter somatic cell counts at dry-off and early lactation

Aldo Dal Prà[1,2Y], Filippo Biscarini[3*Y], G.L. Cavani[4], S. Bacchelli[5], A. Iotti[6], Sara Borghi[7], M. Nocetti[8], Paolo Moroni[7,9]

[1]Centro Ricerche Produzioni Animali (C.R.P.A.) S.p.A., 42121, Reggio Emilia, Italy
[2]Institute of Bioeconomy (IBE), National Research Council, 50145, Florence, Italy
[3]Institute of Agricultural Biology and Biotechnology, National Research Council, 20133, Milan, Italy
[4]Albalat, Società Agricola Cooperativa, 41122, Modena, Italy
[5]Bonlatte, Società Agricola Cooperativa, 41113, Castelfranco Emilia, Modena, Italy
[6]Progeo, Società Cooperativa Agricola, 42122, Reggio Emilia, Italy
[7]Università degli Studi di Milano, Dipartimento di Medicina Veterinaria e Scienze Animali, 26900, Lodi, Italy
[8]Consortium of Parmigiano Reggiano Cheese, 42124, Reggio Emilia, Italy
9Quality Milk Production Services, Animal Health Diagnostic Center, Cornell University, Ithaca, NY 14853, USA

[Y]These authors contributed equally to this work.
*filippo.biscarini@ibba.cnr.it

Table S1: Average DSCC per SCC class (horizontal axis: from 50k to > 1000k) overall and per parity or timepoint, in the top section of the table; proportion of records with DSCC > 70% in the bottom section of the table.

	term	50	100	150	200	250	500	1000	1000+
	overall	43.930	48.725	51.748	55.238	56.020	58.881	61.189	64.815
	parity_2	43.935	48.781	51.314	55.821	56.480	60.687	65.076	66.928
	parity_3	43.412	48.420	51.461	54.513	55.553	57.420	57.633	63.661
avg(DSCC)	parity_4	44.850	49.063	53.806	54.949	55.935	57.652	60.946	63.920
	timepoint_1	45.657	50.037	53.010	55.242	55.483	58.230	59.461	61.892
	timepoint_2	46.147	46.983	49.287	51.215	53.255	57.855	62.111	69.490
	timepoint_3	41.186	50.200	54.478	64.804	64.308	64.782	70.136	71.602
	overall	0.050	0.097	0.133	0.214	0.209	0.263	0.303	0.396
	parity_2	0.046	0.105	0.147	0.245	0.253	0.320	0.422	0.471
	parity_3	0.052	0.078	0.112	0.200	0.177	0.237	0.237	0.348
%DSCC > 70%	parity_4	0.063	0.103	0.128	0.156	0.173	0.186	0.232	0.373
	timepoint_1	0.026	0.069	0.097	0.162	0.143	0.213	0.238	0.277
	timepoint_2	0.072	0.085	0.128	0.168	0.194	0.298	0.364	0.588
	timepoint_3	0.046	0.182	0.275	0.495	0.500	0.461	0.595	0.668
