# Supplementary material for: Relationship between total and differential quarter somatic cell counts at dry-off and early lactation
Source: PLoS One. 2022 Oct 17;17(10):e0275755. doi: 10.1371/journal.pone.0275755 (PMC9576081; doi:10.1371/journal.pone.0275755)
Supplement: S2 Table — (PDF) [file pone.0275755.s002.pdf]

Relationship between total and differential quarter somatic cell counts at dry-off and early lactation

Aldo Dal Prà[1,2Y], Filippo Biscarini[3*Y], G.L. Cavani[4], S. Bacchelli[5], A. Iotti[6], Sara Borghi[7], M. Nocetti[8], Paolo Moroni[7,9]

[1]Centro Ricerche Produzioni Animali (C.R.P.A.) S.p.A., 42121, Reggio Emilia, Italy
[2]Institute of Bioeconomy (IBE), National Research Council, 50145, Florence, Italy
[3]Institute of Agricultural Biology and Biotechnology, National Research Council, 20133, Milan, Italy
[4]Albalat, Società Agricola Cooperativa, 41122, Modena, Italy
[5]Bonlatte, Società Agricola Cooperativa, 41113, Castelfranco Emilia, Modena, Italy
[6]Progeo, Società Cooperativa Agricola, 42122, Reggio Emilia, Italy
[7]Università degli Studi di Milano, Dipartimento di Medicina Veterinaria e Scienze Animali, 26900, Lodi, Italy
[8]Consortium of Parmigiano Reggiano Cheese, 42124, Reggio Emilia, Italy
9Quality Milk Production Services, Animal Health Diagnostic Center, Cornell University, Ithaca, NY 14853, USA

[Y]These authors contributed equally to this work.
*filippo.biscarini@ibba.cnr.it

Table S2: estimates of the fixed effects and variance components from the bivariate mixed model used to estimate the phenotypic correlations between DSCC and log(SCC).

fixed effect	value	s.e.
herd.log_scc.castello	2.9145	0.123
herd.log_scc.Corte	3.1335	0.127
herd.log_scc.oppio	3.7996	0.140
herd.DSCC.castello	44.4846	1.404
herd.DSCC.Corte	44.3293	1.441
herd.DSCC.oppio	45.4850	1.599
timepoint.log_scc.1	1.0098	0.090
timepoint.log_scc.2	1.1851	0.037
timepoint.log_scc.3	0.0000	0.000
timepoint.DSCC.1	5.2846	0.987
timepoint.DSCC.2	3.3321	0.408
timepoint.DSCC.3	0.0000	0.000
parity.log_scc.2	-0.4560	0.060
parity.log_scc.3	-0.1078	0.063
parity.log_scc.4	0.0000	0.000
parity.DSCC.2	-1.0408	0.685
parity.DSCC.3	-1.5831	0.720
parity.DSCC.4	0.0000	0.000

treatment.log_scc.antibiotico	0.8056	0.037
treatment.log_scc.sigillante	0.0000	0.000
treatment.DSCC.antibiotico	3.5726	0.417
treatment.DSCC.sigillante	0.0000	0.000
breed.log_scc.crossbred	-0.1424	0.064
breed.log_scc.holstein	0.0000	0.000
breed.DSCC.crossbred	-1.9697	0.732
breed.DSCC.holstein	0.0000	0.000
DIM.log_scc	0.0029	0.000
DIM.DSCC	0.0063	0.004
yield_tot.log_scc	0.0000	0.000
yield_tot.DSCC	0.0002	0.000
**variance component**	**value**	**s.e.**
cow_nid.log_scc	0.3303	0.02136
cow_nid.log_scc_cow_nid.DSCC	1.5409	0.18569
cow_nid.DSCC	45.355	2.78
Residual.log_scc	1.9036	0.02528
Residual.log_scc_Residual.DSCC	8.607	0.21084
Residual.DSCC	226.05	3.0012
