# Supplementary material for: Relationship between total and differential quarter somatic cell counts at dry-off and early lactation
Source: PLoS One. 2022 Oct 17;17(10):e0275755. doi: 10.1371/journal.pone.0275755 (PMC9576081; doi:10.1371/journal.pone.0275755)
Supplement: S1 Fig — (PDF) [file pone.0275755.s003.pdf]

Relationship between total and differential quarter somatic cell counts at dry-off and early lactation

Aldo Dal Prà[1,2Y], Filippo Biscarini[3*Y], G.L. Cavani[4], S. Bacchelli[5], A. Iotti[6], Sara Borghi[7], M. Nocetti[8], Paolo Moroni[7,9]

[1]Centro Ricerche Produzioni Animali (C.R.P.A.) S.p.A., 42121, Reggio Emilia, Italy
[2]Institute of Bioeconomy (IBE), National Research Council, 50145, Florence, Italy
[3]Institute of Agricultural Biology and Biotechnology, National Research Council, 20133, Milan, Italy
[4]Albalat, Società Agricola Cooperativa, 41122, Modena, Italy
[5]Bonlatte, Società Agricola Cooperativa, 41113, Castelfranco Emilia, Modena, Italy
[6]Progeo, Società Cooperativa Agricola, 42122, Reggio Emilia, Italy
[7]Università degli Studi di Milano, Dipartimento di Medicina Veterinaria e Scienze Animali, 26900, Lodi, Italy
[8]Consortium of Parmigiano Reggiano Cheese, 42124, Reggio Emilia, Italy
9Quality Milk Production Services, Animal Health Diagnostic Center, Cornell University, Ithaca, NY 14853, USA

[Y]These authors contributed equally to this work.
*filippo.biscarini@ibba.cnr.it

Figure S1: 2x2 tables with the distribution of samples along combinations of SCC and DSCC above/below critical thresholds. Green cells refer to the proportion of samples which are likely free of subclinical mastitis (both criteria below the respective critical thresholds); red cells indicate the proportion of samples which likely have subclinical mastitis (both criteria above the respective critical thresholds). Yellow cells indicate samples which may require a second look. Three different thresholds for SCC have been used (200k, 150k, 100k).

	SCC > 200k	
dscc_class	no	yes
<70	65.50	20.16
70+	5.51	8.84

	SCC > 150k	
dscc_class	no	yes
<70	61.93	23.72
70+	4.54	9.80

	SCC > 100k	
dscc_class	no	yes

<70	55.45	30.20
70+	3.58	10.76
